# Psychotic Symptoms Following Subthalamic DBS in Parkinson's Disease: Three Clinical Cases and Literature Review

**DOI:** 10.1002/ccr3.72666

**Published:** 2026-05-10

**Authors:** Saeed Abdollahifard, Raziyeh Rezaei, Erfan Sanaei, Fatemeh Asadi, Amirmohammad Farrokhi, Malihe Mehdinejad Haghighi, Fatemeh Khademi, Sina Sabet, Aliasghar Karimi, Ali Razmkon, Nematollah Jaafari

**Affiliations:** ^1^ Research Center for Neuromodulation and Pain Shiraz University of Medical Sciences Shiraz Iran; ^2^ Student Research Committee Shahid Sadoughi University of Medical Sciences Yazd Iran; ^3^ Faculty of Medicine Bushehr University of Medical Sciences Bushehr Iran; ^4^ Trauma Research Center, Shahid Rajaee (Emtiaz) Trauma Hospital, Shiraz University of Medical Sciences Shiraz Iran; ^5^ Unité de Recherche Clinique Intersectorielle en Psychiatrie à Vocation Régionale Pierre Deniker Centre Hospitalier Henri Laborit Poitiers France

**Keywords:** deep brain stimulation, functional neurosurgery, neurosurgery, Parkinson's disease, psychotic symptoms

## Abstract

Psychosis may emerge after subthalamic deep brain stimulation in Parkinson's disease, regardless of prior psychiatric history. Early recognition of stimulation‐related behavioral changes, careful dopaminergic adjustment, and multidisciplinary postoperative monitoring are essential to differentiate DBS‐induced psychotic symptoms from disease progression and improve neuropsychiatric and functional outcomes.

## 1 Introduction

Deep brain stimulation (DBS) is a well‐established neurosurgical therapy for advanced Parkinson's disease (PD), typically reserved for patients with medically refractory motor fluctuations or dyskinesias [1]. High‐frequency stimulation of basal ganglia targets (most commonly the subthalamic nucleus or globus pallidus internus) can markedly improve cardinal PD motor symptoms, often reducing medication requirements [2]. In carefully selected patients, DBS has proven safe and effective for these indications [1], and its use has become widespread as a second‐line treatment option when medications fail to control symptoms [1, 2]. Despite these benefits, DBS can cause a spectrum of neuropsychiatric side effects. Reported complications include mood and behavioral changes such as depression, (hypo)mania, anxiety, impulsivity, apathy, and in rare cases, suicidal ideation [3, 4]. Psychotic symptoms (visual hallucinations, delusions, delirium) have also been observed postoperatively. For example, Hanna et al. describe an acute‐onset psychotic episode with suicidality and depression following pallidal DBS [3]. The mechanisms underlying these effects are not fully understood, but hypotheses include “microlesion” effects from electrode placement and dysregulated stimulation of limbic circuits [4]. In one recent systematic review of STN‐DBS patients, the most common postoperative psychiatric disorders were depression, mania, and anxiety, with psychosis noted alongside these in multiple reports [4]. Psychosis after DBS appears relatively uncommon but clinically significant. Older age at implantation was associated with a higher risk and earlier onset of psychosis. However, psychotic symptoms may also emerge as part of the natural course of PD or as medication‐induced phenomena, making attribution to DBS challenging [5]. In this context, individual case reports remain valuable for enhancing the understanding of DBS‐related psychiatric complications. The case presented here—PD patients developing new‐onset psychosis after DBS—contributes to the literature by illustrating this rare but serious outcome. By documenting the clinical features, timing, and response to DBS parameter adjustments and psychotropic treatment, we aim to shed light on potential mechanisms and emphasize the need for vigilant neuropsychiatric monitoring in DBS recipients. This report highlights gaps in knowledge regarding the causation of post‐DBS psychotic symptoms and underscores the importance of further study in this area.

## 2 Case History/Examination

Patients with medically refractory PD referred to the Neuromodulation and Pain clinic in Shiraz, Iran, for Subthalamic Nucleus Deep Brain Stimulation (STN‐DBS) who developed psychotic symptoms were isolated from our database and called for a visit to our clinic. Patients were interviewed for evaluation of mental status and possible causes of recently developed symptoms by a team consisting of a neurosurgeon, a psychiatrist, and a neurologist. The details of the surgical intervention methods were previously published by Razmkon et al. [6]. In addition to the brief description of each case, data on the Unified Parkinson's Disease Rating Scale (UPDRS) score for each patient and the last follow‐up stimulation parameters are provided in Tables 1, 2, 3. All patients underwent routine preoperative neurological and psychiatric screening, including structured psychiatric interviews and cognitive assessment, and none met criteria for active psychosis at the time of surgery. The race of all reported patients was Middle Eastern.

**TABLE 1:** Summary of presentation of PD patients with psychotic symptoms.

Cases	Onset of psychotic symptoms after surgery	Psychiatric symptoms	Medications
1	First week	Hallucinations delusions illusions, paranoia, homicide, suicide	Levodopa/benserazide (250 mg/day), clozapine (50 mg/day), donepezil (20 mg mg/day), mirtazapine (30 mg/day)
2	3 months	Delusion, hallucination, suicide attempt	Clozapine (25 mg/day), imipramine (75 mg/day), citalopram (40 mg/day), trifluoperazine (1 mg/day)
3	1 month	Delusion	Quetiapine (150 mg/day), clozapine (50 mg/day), donepezil (10 mg/day)

### 2.1 Case 1

A 58‐year‐old female patient with histories of Generalized Anxiety Disorder (GAD) and obsessive‐compulsive disorder (OCD) showed significant levels of dyskinesia prior to undergoing STN‐DBS (subthalamic nucleus deep brain stimulation). Prior to surgery, the patient had exhibited mild cognitive impairment (UPDRS‐I = 26), overcoming hallucinations, mild depression, and paranoid behavior, most likely due to being on high doses of levodopa.

On the first week after she received her DBS stimulation, the patient experienced increased cognitive and sleep disturbances, visual and auditory hallucinations, as well as delusions/illusions. This resulted in the patient becoming paranoid, expressing suicidal/homicidal ideation. At the initiation of treatment with levodopa/benserazide (250 mg/day) and clozapine (50 mg/day), she was subsequently placed on donepezil (10 mg every 12 h) and mirtazapine (30 mg/day).

### 2.2 Case 2

The 53‐year‐old male patient experienced persistent tremors and eventually dyskinesia with obsessive behaviors prior to STN‐DBS; he had a very severe level of impairment with motor functioning pre‐operatively (UPDRS‐III = 77). He developed depression, paranoia, visual hallucinations, and delusions of control 3 months after undergoing surgery. His initial treatment plan consisted of clozapine (12.5 mg/day), donepezil (4 mg/day), and clonazepam (1 mg/day). He made a suicide attempt 8 months after his surgery, and his medication regimen was then changed to clozapine (25 mg/day), imipramine (75 mg/day), citalopram (40 mg/day), and trifluoperazine (1 mg/day).

### 2.3 Case 3

**FIGURE 1:**
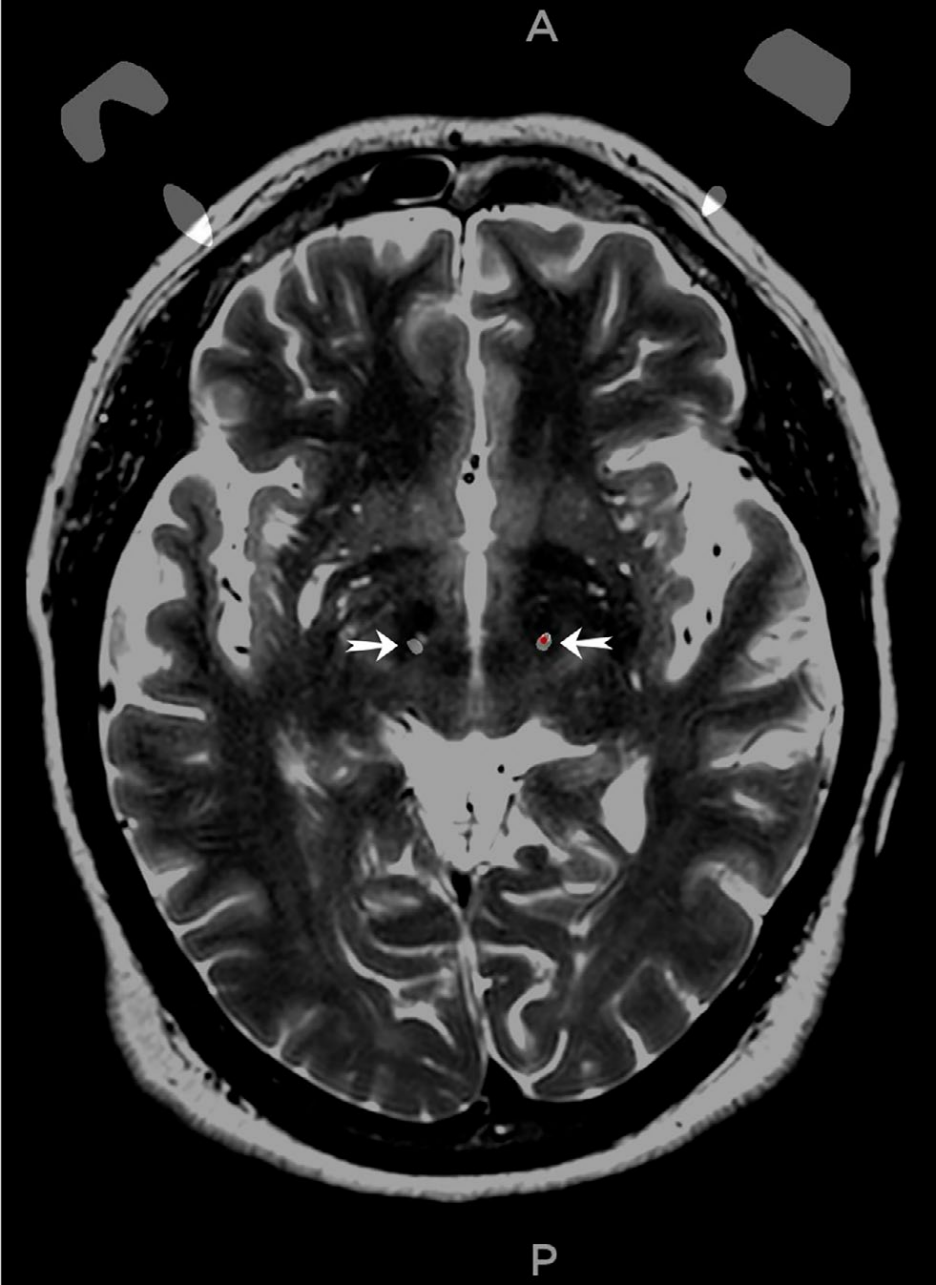
The merged T2 sequence of magnetic resonance imaging and post‐op computed tomography scan of the patient indicates the location of leads. The pointer in each site indicates the location of the lead.

A 56‐year‐old woman with bradykinesia‐dominant PD and no prior psychiatric history underwent STN‐DBS. One month postoperatively, she developed delusions and motor difficulties, including swallowing problems and limb numbness. Postoperative CT confirmed proper lead placement (Figure 1). Her treatment included quetiapine (150 mg/day), clozapine (50 mg/day), and donepezil (10 mg/day), and the device was subsequently turned off due to symptom severity.

## 3 Differential Diagnosis

When a patient develops an acute or subacute onset of psychotic symptoms after being implanted with the STN‐DBS system, there are many factors that must be considered. DBS‐induced psychotic symptoms can be attributed to the timing of when the stimulation was first started, and when a patient develops these psychotic symptoms, the type of hallucinations and delusions that are present, and the presence of suicidal or homicidal thoughts. Most importantly, patients experiencing psychotic symptoms following STN‐DBS implant have been diagnosed with Parkinson's Disease Psychosis (PDP). Generally, PDP develops slowly over time in patients with severe Parkinson's Disease and involves a decline in cognitive function and an increase in the amount of dopaminergic therapy given for treatment. Most of the time, patients begin to develop visual hallucinations followed by the development of delusions over the course of their disease. Although it is possible to develop postoperative delirium, the presence of persistent, specific, and organized themes related to the psychotic features makes this diagnosis less likely. The possibility of medication‐induced psychosis was also considered; however, the persistence of symptoms despite stable dopaminergic therapy suggests otherwise. Finally, primary psychiatric disorders may predispose patients to psychosis, but the acute presentation in patients without prior psychiatric history (Cases 2 and 3) supports a DBS‐related etiology. Collectively, these observations suggest that DBS stimulation acted as a precipitating or exacerbating factor in the development of psychotic symptoms.

## 4 Outcome and Follow‐Up

In Case 1, the combination of clozapine, donepezil, and mirtazapine managed the patient's psychotic symptoms. The patient's hallucinations and other non‐motor symptoms were controlled 6 months after the surgery, and medications were gradually reduced. Case 2 began treatment with low‐dosage clozapine and additional medications. However, the patient's medicine needs increased after he attempted suicide. Two years later, he reported that all psychotic and depressive symptoms were gone and that he returned to the workplace. Case 3 showed that motor and psychotic symptoms did not improve after medication, and DBS stopped working. The patient stayed in bed until he died 2 years after his surgery. UPDRS scores before and after his surgery are shown in Table 2 and indicate improvement in motor function, but inconsistent results in non‐motor functioning.

**TABLE 2:** UPDRS score of patients comparing before and after surgery.

Cases^a^	UPDRS‐I	UPDRS‐II	UPDRS‐III	UPDRS‐IV
1	26/9	26/4	63/18	14/1
2	19/10	19/10	77/25	17/0
3	28/35	22/36	62/58	11/10

^a^ Values are presented as preoperative/postoperative scores.

**TABLE 3:** The last stimulation parameters of patients.

Cases^a^	Stimulation amplitude (R/L)	Pulse width (R/L)	Stimulation frequency (R/L)
1	3.4/4	60/60	80/80
2	3.8/3.2	60/60	125/125
3	Not available	Not available	Not available

^a^ Values are presented as preoperative/postoperative scores.

## 5 Discussion

PD is the second most prevalent neurodegenerative disease, having the fastest growing prevalence according to the Global Burden of Disease Study 2015. Projections estimate people living with Parkinson's disease to be 25.2 million [7]. Noted by loss of dopaminergic neurons in the substantia nigra, PD proposes a wide array of disabilities affecting physiological and psychological function [8]. Though the most noticeable symptoms of PD are motor symptoms, including tremors and bradykinesia, a meta‐analysis by Burchill et al. has demonstrated the detrimental effect of psychiatric symptoms on a patient's current quality of life and future burdens such as cognitive impairment [9]. In this large study, psychosis was highly associated with cognitive impairment and worse disease progression across 21 studies consisting of 159,438 patients. Other effective factors were depression and apathy, which contributed to cognitive impairment. Depression was also associated with disability.

In addition to conventional pharmacotherapy, DBS has been an integral part of the management of patients with PD, mainly targeting the subthalamic nucleus and the globus pallidus [10]. Recent studies have shown an overall superiority of DBS and device‐based interventions in general compared to medical therapy, with a review by Farooqi et al. showing higher benefits in patients with DBS [11, 12]. Though direct electrical stimulation of nuclei corresponding to symptoms may appear as a direct solution to the cause of the symptoms, it is not free of adverse effects. These effects range from short‐term complications, such as surgical complications and postoperative delirium, to long‐term outcomes. Short‐term complications, though more acutely affecting the patient's health, have a low incidence and mostly resolve quickly. Long‐term outcomes prove to be harder to evaluate thoroughly but could have profound effects on patient quality of life; these include cognitive dysfunction, recurrence of symptoms, or infections [13]. On the other hand, brain stimulation therapies, including DBS, have been proven to be effective in cognitive and psychiatric disorders in addition to movement disorders by an umbrella review by Zu et al., though DBS was used mainly for treating the movement disorder rather than the other aspects [14]. However, in another review by Eliufoo et al. it was indicated that though DBS can improve motor symptoms as its aim, cognitive and psychological side effects may be neglected or at least not be addressed accordingly. Studies addressing this issue are not homogenous and a collective recent review may be required to accurately evaluate how these phenomena occur [15]. While DBS is associated with a wide range of neuropsychiatric effects, the present discussion focuses specifically on psychosis as a distinct and clinically significant postoperative complication.

Several studies have evaluated the occurrence of psychiatric diseases, specifically after DBS surgery. Depression is the most frequently reported disease after DBS, being attributed to several factors, including predisposing factors before surgery, direct action on the dopaminergic pathway, and inhibition of specific STN regions or adjacent structures such as the medial forebrain bundle [4]. Depression could also be associated with other disorders such as psychosis, suicidal ideation, and anxiety in patients undergone DBS [16]. Another meta‐analysis of mood effects by Cartmill et al. further emphasizes the ambiguity of this subject, showing the simultaneous occurrence of depression as well as a decline in depression symptoms after DBS surgery, which could have been attributed to different causes such as reduction of levodopa dosage, social factors, etc. This fact, plus the co‐occurrence of depression with other diseases such as anxiety [17]. Altogether, mood disorders have been widely reported after DBS and may increase vulnerability to more severe psychiatric decompensation [18]. However, these manifestations alone do not explain the acute or subacute onset of psychosis observed in some patients following DBS, and we believe that they are therefore considered contributory rather than causative in the present cases.

Also, another study by Erdem et al. showed that DBS on STN may aggravate psychiatric disorders such as depressive episodes requiring antidepressant treatment and impulse control behaviors [19]. A “witty” thesis by Witt et al. was that insertion of a foreign technological device may challenge identity definition and cause psychological issues [20]. On another note, by Mosley et al., DBS could propose an acute insult to the disrupted anatomic and physiologic circuits involved in PD, further causing dissociation between multi‐faceted outcome measures of physical and psychological function [21]. Wilt et al. proposed a conceptual framework exploring how DBS may influence patients' subjective sense of identity and personality through interactions between neurostimulation, disease burden, and psychosocial factors. Their work is primarily theoretical and does not provide direct clinical evidence linking DBS to psychosis. Nevertheless, it highlights the importance of considering patients' psychological experience when evaluating neuropsychiatric outcomes after DBS [22].

Regarding suicidal behavior and ideation, a review was performed by Costanza et al. to further explore the routes through which DBS could contribute to such side effects. Their review illustrated several studies that demonstrate higher suicidal behavior and ideation in patients after DBS. Several hypotheses were introduced explaining this finding, which were connected to dysfunctions in neuroanatomical circuits, specifically based on a dopaminergic system, which could also lead to impulse control disorders [23, 24]. Altogether, in these cases, suicidal thoughts came together with psychotic symptoms, which point to a serious breakdown in psychiatric stability rather than just a mood problem.

Another factor was the involvement of the amygdala in DBS, which could occur due to off‐target stimulation. STN connectivity with frontal cortical regions and prefrontal cortical areas, and disturbance of mood based on these interferences, were also documented in several studies [25, 26]. Another hypothesis was the immunological activation following DBS, leading to multiple immunological pathways leading to behavioral problems. However, clinical data to support the latter thesis were scarce, and more focused studies on patients with DBS were recommended to further assess this [27, 28]. In a different study, Seritan et al. reviewed a total of 82 patients to discover that nine patients developed elevated mood after DBS, occurring during or shortly after programming changes. All patients with elevated mood were males with STN‐DBS and had previous psychiatric comorbidities, though different. Findings from this study were in line with studies by Voon et al., Mosley et al., and Schilbach et al. [29, 30, 31]. Though these elevated mood occurrences are reported across several studies, identification and evaluation of risk factors and DBS parameters related to them are not yet studied in an exact manner [32].

Apathy has also been reported following STN‐DBS, although its etiology remains debated. A review by Vachez et al. on animal models suggests that stimulation of the subthalamic nucleus may influence motivational and reward‐related circuits. This event consistently decreased reward motivation, seeking, and consumption in animal models independent of STN lesions, pointing to the effect of stimulation. Nevertheless, applying these findings to humans has its limitations. Since this study was based on animal models and scalable standard trials were performed, postoperative apathy is often difficult to disentangle from disease progression, reduction of dopaminergic medications, or pre‐existing non‐motor symptoms [33]. Another deduction is that this general decrease in the reward circuits could secondarily lead to dysfunction in other aspects, such as depression or altered cognitive function, in addition to its direct result, apathy [34].

Among psychiatric complications, psychosis represents a distinct and particularly severe outcome following DBS [35]. Psychosis is another rather more serious adverse event occurring after DBS. This can be described as an exacerbation of other psychiatric conditions, happening as a severe form of already existing pathologies or acutely onset episodes. Some studies have mentioned psychosis occurring even before the beginning of stimulation [3, 36, 37]. Since these events occurred before stimulation, it could put some emphasis on the effect of the implantation of electrodes rather than electrical stimulation. One of the novel points addressed by McLean et al. is the “insertional effect,” which is the effect of the insertion of the electrodes without electrical stimulation on the brain. This effect has been shown to have both positive and negative effects on motor and psychological function and similarly requires further clinical and molecular investigation [38]. Another hypothesis is the dysregulation of dopamine release and limbic side effects, as explored before, in the onset of another psychological phenomenon in a more severe manner. The severity of motor function and younger age were seen to be risk factors for the onset of psychosis as well [39]. However, no significant factors such as medicine use or stimulation parameters have been observed to certainly alleviate or worsen the psychosis symptoms so far. Psychosis also poses another risk, further complicating the management process, which is the use of antipsychotic medicine that may worsen the symptoms associated with PD [40]. A finding in the study by Widge et al. was that a brief course of high‐potency antipsychotics could be safe in the treatment of such episodes [37]. The controversy persists regarding psychosis as well, since some studies suggested that higher age is an independent risk factor for the development of psychosis symptoms [5, 41]. Another noteworthy finding of the study by Qureshi et al. was that the lifetime incidence of psychosis in PD patients after DBS was 28.3%, proving that despite popular belief, the incidence of psychosis may not be as low as expected, and their existence may have been neglected [5]. Similar to other symptoms, reduction of dopaminergic drugs showed improvement both in motor and psychological function, though there was a possibility of short‐term exacerbation, happening only temporarily.

Though there have been numerous case reports regarding the occurrence of psychosis in addition to other psychological symptoms in PD patients following DBS, the results of these studies were mostly speculative. They hypothesized based on findings from each study's design and protocol. There have been very limited studies aiming to systematically tackle this issue, though the sequela can range from discomforting to severely disabling in patients. The development of accurate studies with definitive protocols to evaluate these phenomena is necessary since it can encompass pre‐existing conditions exacerbating after DBS or independent of DBS, and newly onset psychological problems. The depth of the matter can also range from molecular insults in electrode placement, tissue stimulation and alterations, psychosomatic occurrences, and pharmacological reactions to changes in medicine dosage and purely psychological events. As is evident, too many factors have already been reported to impact this topic, and further multidisciplinary studies are required to distinguish the key factors. On another note, Memon et al. have discovered multiple disparities regarding the use of DBS, which require further evaluation and studies to accurately address them, especially among vulnerable populations, which can indicate deeper psychosocial elements in play [42].

This study suggests that the mentioned psychotic features were more associated with stimulation than with Parkinson's disease itself. The post‐operative psychosis following DBS should be considered as a pathophysiologically separate entity from PDP because it varies in terms of initiating factors, timing of onset, and clinical course. PDP generally develops insidiously in the context of advanced disease, cognitive decline, and extended exposure to dopaminergic medications, with visual hallucinations usually preceding delusions. The primary contributing factor is exposure to PD medications, especially dopamine agonists and higher doses of levodopa [43]. In contrast, DBS‐induced psychosis usually appears within a short period after electrode implantation or stimulation begins. Additional factors that may play an integral role in this condition include direct current spread into limbic/associative circuits of the subthalamic nucleus, microlesion effects, perioperative delirium, abrupt medication adjustments, and maladaptive network plasticity. Unlike PDP, these episodes may vary with stimulation settings or resolve after reprogramming of stimulation or psychiatric treatment, suggesting a causal link to DBS itself [44, 45]. Risk modifiers also vary; PDP is strongly linked to dementia and prolonged medication use, while DBS complications are associated with younger age at surgery, preexisting psychiatric conditions, and specific lead trajectories [4]. Clinically, PDP develops gradually and typically starts with visual phenomena, whereas DBS‐related psychosis can include sudden paranoia, delusions of control, suicidality, and severe emotional disturbances that appear abruptly and may resolve with device adjustments [3].

Overall, the sudden or subacute onset, temporal clustering around DBS initiation, and behavioral modulation by stimulation suggest stimulation‐induced psychotic symptoms, setting these episodes apart from the gradual, medication‐related course of postoperative dopamine dysregulation. Together, these observations strengthen the case that psychosis after DBS is mechanistically distinct and reinforce the need for solid psychiatric screening, careful perioperative dopaminergic management, and individualized programming strategy to lessen risk.

To sum up, we have reported three cases of patients with PD who developed psychotic symptoms that are suggested to be caused by DBS stimulation. Since findings regarding psychiatric sequela after stimulation are mainly hypothetical and circumstantial, with limited literature on the subject, larger‐scale systematic studies are needed to investigate them further. It is also possible that optimization of medical therapy prior to surgery may have mitigated psychiatric risk in certain patients, particularly those with subtle preoperative neuropsychiatric symptoms.

## 6 Conclusion

Following STN‐DBS treatment, psychotic symptoms are classified as a separate clinical entity from PDD. Typically, they may begin to manifest within weeks to months after the initial onset of stimulation. These symptoms may present as delusions, paranoia, or even suicidal ideation and may respond well to reprogramming of the DBS device and through therapy. Pre‐existing cognitive impairment, history of psychiatric illness, younger age, and specific lead trajectories are all risk factors for developing psychotic symptoms following STNB‐DBS. Important steps in minimizing the risk of developing psychotic symptoms following STNS‐DBS include careful preoperative psychiatric evaluation/assessment and individualized programming of the DBS device postoperatively, and close monitoring for patients after surgery. Although STN‐DBS has been shown to improve a patient's motor function, the identification and management of severe neuropsychiatric complications associated with STN‐DBS treatment are critical to achieving optimal outcomes for patients.

## Author Contributions

**Saeed Abdollahifard:** conceptualization, data curation, formal analysis, methodology, validation, writing – original draft, writing – review and editing. **Raziyeh Rezaei:** data curation, investigation, methodology, validation, writing – original draft, writing – review and editing. **Erfan Sanaei:** data curation, resources, visualization, writing – original draft, writing – review and editing. **Fatemeh Asadi:** methodology, validation, writing – original draft, writing – review and editing. **Amirmohammad Farrokhi:** formal analysis, investigation, validation, writing – original draft, writing – review and editing. **Malihe Mehdinejad Haghighi:** data curation, supervision, validation, writing – original draft, writing – review and editing. **Fatemeh Khademi:** conceptualization, methodology, validation, writing – original draft, writing – review and editing. **Sina Sabet:** formal analysis, investigation, validation, writing – original draft, writing – review and editing. **Aliasghar Karimi:** conceptualization, data curation, methodology, validation, writing – original draft, writing – review and editing. **Ali Razmkon:** conceptualization, methodology, supervision, validation, writing – original draft, writing – review and editing. **Nematollah Jaafari:** conceptualization, investigation, methodology, supervision, validation, writing – original draft, writing – review and editing.

## Funding

The authors have nothing to report.

## Disclosure

This manuscript was presented at a national congress in Iran, but there is no link available for the congress presentation.

## Ethics Statement

This study was performed in accordance with the Declaration of Helsinki. This human study was approved by the Ethics Committee of Shiraz University of Medical Sciences—approval: IR.SUMS.REC.1403.234. All adult participants provided written informed consent to participate in this study. Written informed consent was obtained from the individual(s) for publication of the details of their medical case and any accompanying images.

## Consent

Informed consent was obtained from all individual participants included in this study.

## Conflicts of Interest

The authors declare no conflicts of interest.

## Data Availability

All data generated or analyzed during this study are included in this article. Further enquiries can be directed to the corresponding author.
